# From the periphery to inclusion within the health system: promoting community health worker empowerment as a way forward

**DOI:** 10.1186/s12875-024-02523-0

**Published:** 2024-07-26

**Authors:** Linnea Stansert Katzen, Steve Reid, Christina Laurenzi, Mark Tomlinson

**Affiliations:** 1 Institute for Life Course Health Research, Department of Global Health, Faculty of Medicine and Health Sciences, Stellenbosch University, 6023 Clinical Building, Francie van Zijl Drive, Tygerberg Campus, Cape Town, Tygerberg, 7505 South Africa; 2 Swedesd, Department of Women’s and Children’s Health, Uppsala University, Uppsala, Sweden; 3 Department of Family, Community and Emergency Care, Faculty of Health Sciences, University of Cape Town, Rondebosch, South Africa; 4 School of Nursing and Midwifery, Queens University Belfast, Belfast, UK

**Keywords:** Community health workers, Empowerment, Primary health care, Inclusion, South Africa

## Abstract

**Background:**

Community health worker programmes have the potential to contribute critically towards universal health coverage. However, CHWs globally have often continued to operate on the periphery of the health care system, viewed as a non-essential cadre. This results in a workforce that often remains disempowered and under-supported. This paper presents evidence from a study conducted in a rural part of South Africa, to better understand issues of CHW prioritisation, integration, and empowerment.

**Methods:**

We applied an analytical lens based on empowerment theory and conducted a secondary analysis of qualitative data emerging from a sub-study of a cRCT evaluating the effectiveness of supportive supervision for CHWs within a large-scale national CHW programme. The cRCT was conducted between 2017 and 2022, and 39 CHWs were included in the study.

**Results:**

We organised our findings across the four domains of structural empowerment; information, resources, support, and opportunity, and mapped these domains against the domains of psychological empowerment. Our findings show how CHWs are still working in the periphery of the healthcare system. Without sufficient prioritisation, high level-support from national and district governments, and sufficient investments in programmatic domains—such as training, equipment, and supportive supervision—it is likely that the CHW cadre will continue to be seen as informal health care workers.

**Conclusions:**

CHW empowerment could be a lever to potentially transform the current health system towards universal coverage; however, this process can only happen with sufficient high-level prioritization and investment.

**Supplementary Information:**

The online version contains supplementary material available at 10.1186/s12875-024-02523-0.

## Introduction

Deploying community health workers (CHWs) in low- and middle-income countries (LMICs) is an important strategy to bridge service delivery gaps in health care, and support healthcare system functioning [1–3]. In LMICs, this has resulted in significant improvements in population-level health contributing to achieving the Sustainable Development Goals (SDGs) [4, 5]. Community health worker rogrammes have the potential to be pivotal in contributing to universal health coverage. Many programmes however struggle to maintain effectiveness when taken to scale [6]. There are persistent challenges linked to how CHW programmes are structured and implemented, and how resources are allocated to them. Insufficient training, lack of equipment, and fragmented supervision systems are some of the most pressing programmatic challenges [7].

Importantly, these challenges are also linked to their perceived legitimacy and the political will to support them. Despite a convincing investment case put forward by the Centre for Accelerating Innovation and Impact [8] CHWs have continued to operate on the periphery, rather than playing a central role, in the health care system, often viewed as a non-essential cadre until governments need to respond nimbly in times of crisis. In South Africa for example, despite a long history of a community health workforce, the transition to a democratically elected government in 1994 and the implementation of a new primary health care plan resulted in a shift away from CHWs and a focus on doctors and nurses [9]. However, with the increasing health and social burdens posed by the emerging HIV epidemic, community-based care workers emerged as a critical means of support. A CHW programme was reinstated, predominantly through Non-Governmental Organisations (NGOs) and community partnerships [10, 11]. The COVID-19 pandemic also ushered in a new urgency around how to operationalize CHWs, who were seen as essential for contact tracing and vaccine education and advocacy [12].

Globally, differences in how CHW programmes are prioritized, has resulted in a patchwork of CHW programmes, administered and implemented by both NGOs as well as governments at national and provincial level [13]. Less visible but more protracted crises within healthcare systems—linked to healthcare worker shortages or funding freezes—have also prompted increased task-shifting or “task-dumping” where CHWs are expected to take on an increasing number of roles and responsibilities [14]. Rather than conferring a sense of legitimacy or respect to the CHW role, task shifting has often reduced the effectiveness of CHWs through maintaining ‘vertical’ programmes separate to services in health facilities. The World Health Organization [15] sees CHWs as an integral part of multi-disciplinary teams, and note that:

Connecting community health workers with facility-based staff is a particularly important aspect, both to improve the quality of care offered by the former and because they can play a vital role in linking communities to facilities and delivering population-based services.

The question of how to effectively harness and integrate CHWs into well-functioning health systems is a complex problem [16]. To date, most solutions have focused on remuneration, training, and role definition. There are examples of successful integration, where CHWs have been conceptualized as part of the broader public health agenda, and deliberately embedded within health systems [17]. Community-oriented primary care (COPC) provides a useful conceptual framework for their integration [18]. In the Brazilian Family Health Team model for example, each outreach team includes a physician, a nurse, a nurse assistant and a variable number of CHWs, depending on the size and vulnerability of the population served [11]. This approach enables health teams to provide both preventative and curative health care, particularly in underserved areas [17, 19].

Despite some evidence of success persistent issues include the resourcing, legitimacy, and integration of CHWs into the health system [6]. While NGO-administered CHW programmes may confer certain benefits—for instance, greater capacity for training and supervision—there are also limitations to the credibility and the collaborative relationships that these CHWs may be able to develop. This problem is largely cyclical. Because CHWs in many settings are not seen as central actors in a functional health system, they tend to be poorly resourced, and not widely viewed as legitimate [20]. This results in a workforce that has significant potential to support and mitigate health inequities, yet remains disempowered, under-supported, and in a weak bargaining position.

In South Africa, these contradictions exist alongside social, economic, and political complexities. In the context of a highly inequitable, fragmented health system, most South Africans still struggle to access quality health care, and often even the most basic care provision is inconsistent and inadequate [21]. Given this context, CHWs at scale could expand the reach of the government health system. Coupled with adequate supervision and support, they could greatly enhance the quality of care as well as the ability of the health system to respond to national health priorities proactively. The past decade has seen a shift in how CHWs are prioritized in South Africa. Following the Brazilian model, a revised national CHW program was introduced in 2011 and Ward-Based Outreach Teams (WBOTS) [11] were established, that pair a community health approach with the strengthening of primary care services [22–24]. This is also part of a strategy to implement National Health Insurance (NHI) to achieve universal health coverage and ensure access to health care for all [25]. The structure of these programmes in South Africa varies between the provinces, for example, in the Eastern Cape (where this study took place), CHWs are directly employed by the Eastern Cape Department of Health. In the Western Cape on the other hand, there is a government–NGO partnership where NGOs are contracted to implement CHW programmes. Both models have strengths and limitations, and in terms of inclusion in the health care system, it can be argued that CHWs employed by NGOs may be even further from inclusion, not being employed by the DoH, while in fact often conducting better quality work. The WBOT teams work at the ward level (sub-division of a municipality), where a group of six to ten CHWs are directly linked to a primary healthcare facility and are supervised by primary healthcare clinic managers and operational team leaders. CHWs in the WBOT teams are government-employees and receive a salary, and while they thus are formally linked to health care sector, the cadre is often seen as not full members of the health care workforce. While these shifts reflect a recognition of the importance of multi-disciplinary care, there are still significant gaps in how CHWs are operationalized, supervised, and valued. Recent evidence [26] has illustrated the absence of CHW voices in both the programmatic and research evaluations of programmes.

This paper presents evidence from a study conducted in a rural part of South Africa, to better understand issues of CHW prioritisation, integration, and empowerment.

## Methodology

We applied an analytical lens based on empowerment theory and conducted a secondary analysis of qualitative data emerging from a large-scale CHW programme in the Eastern Cape Province of South Africa. These data, analysed first in three linked publications [26–29] were part of a qualitative sub-study embedded within a larger cluster randomised trial (cRCT), called the Eastern Cape Supervision Study [ECSS] [29, 30]. The ECSS trial, conducted in 2017–2022, investigated whether quality supervision and support provided to government CHWs improve maternal and child outcomes compared to routine supervision delivered by the primary healthcare system. In the study, 37 CHWs from eight clinics, all employed by the Eastern Cape Department of Health, were trained in an NGO model (Philani) previously shown to be effective [31, 32]. The NGO model is a home visiting intervention program for maternal and child health. Mentor Mothers are CHWs recruited from the areas where they live and whom deliver home visits and provide support to mothers during pregnancy and during the child’s first five years. Training was conducted over four-weeks focusing on maternal and child health, followed by a two-week period shadowing experienced CHWs (Mentor Mothers) in the field [29]. The clinics were then randomised into control and intervention groups. CHWs from clinics in the control carried on working as previously, with whatever routine supervision they normally received. CHWs from intervention clinics received a supportive package of supervision provided to them at least bi-weekly in the field, additional equipment, and transport. The results of the intervention tested in the cRCT showed no statistically significant differences, however benefits were observed for 4 outcomes: increasing breastfeeding for 6 months, reducing malnutrition, increasing ARV adherence, and improving developmental milestones.

The qualitative sub-study, conducted between 2018–2022, aimed to understand CHW experiences of their work as CHWs, the CHW programme in general, and of the intervention implemented in the parent trial. The interview guides used are provided in the supplementary files.

### Theoretical framing

**Fig. 1:**
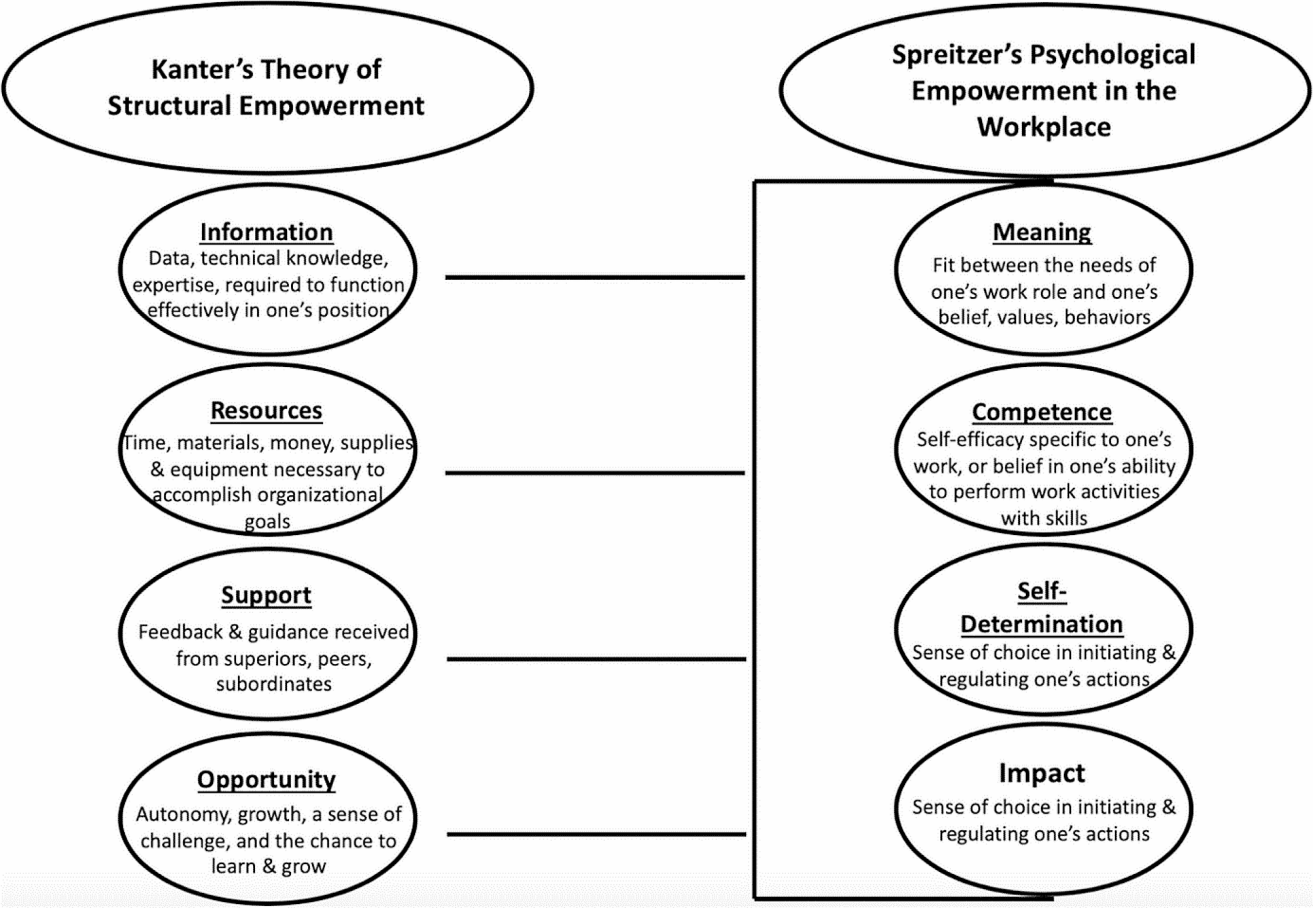
Empowerment framework [33]

Empowerment theory has been used extensively in healthcare research. The domains of both structural empowerment and psychological empowerment, and how these relate to each other, are outlined in Fig. 1 below [33]. It has been established that there is a clear relationship between the domains of structural empowerment and psychological empowerment; fulfilling domains for structural empowerment leads to psychological empowerment, which is closely linked with job satisfaction and performance [34]. Heather and colleagues [35] have argued that structural empowerment leads to psychological empowerment and job satisfaction over time. Investments in empowerment can have longstanding effects on health workforce motivation and retention. Spreitzer [36] argues that psychological empowerment is the response to empowering practices and conditions through which employees perceive their work as being meaningful and having impact. Drawing on frameworks for structural and psychological empowerment, we identify critical thematic areas where gaps and prospects for CHW empowerment emerged from our data.

### Sample

For this qualitative sub-study, 24 CHWs were interviewed. For the first round of interviews (outlined below in Table 1), we used criterion sampling, that is, sampling based on a pre-established criterion, in this case being a CHW in the intervention arm of the trial [37]. All CHWs allocated to the intervention arm of the trial were offered an opportunity to participate in the study [26]. Participants had all been working as CHWs for varying periods of time prior to the implementation of this study, ranging from 13 months to 24 years. Of the 18 CHWs included in the first set of interviews and focus groups 16 were female and two were male, and ages ranged between 25 and 58. Of the 16 CHWs interviewed in second round of interviews, 15 were female and one was male. Ages ranged between 39 and 59 years. Supervisors (*n* = 2) were both female and had been working as supervisors for five years prior to the cRCT starting. All CHWs were based in the rural Eastern Cape and were from a relatively disadvantaged background. Education levels varied from Grade 5 to postgraduate diplomas and all CHWs were literate. For the stakeholder interviews, we recruited from eight governmental clinics that were part of the parent trial, with four clinics having received the enhanced-supervision programme through the trial. Stakeholders were in this case defined as key informants involved in the government implemented CHW programme in the study area and/or in the parent trial [28]. Stakeholders were involved on different levels; clinic personnel were either operational managers (clinic managers of the governmental primary healthcare clinics included in the cRCT) or outreach team leaders (government employed CHW supervisors). cRCT supervisors were employed by the NGO responsible for the implementation of the supervision package. Lastly, for the second round of interviews, we interviewed eight CHWs from each of the two arms of the parent trial, and two supervisors from the intervention arm individually (total *n* = 18). CHWs were purposefully sampled to ensure representation from each of the study clinics. However, within the pools of CHWs at each clinic, random sampling was used, and CHWs from each of the study clinics were randomly drawn from a hat and approached for interviews [27].

**Table 1:** Overview of participants

Participant type and *N*	Number of clinics	Data collection
24 CHWs	8	Individual interviews
18 CHWs	4	FGDs
9 Operational Managers and Team leaders	8	Individual interviews
4 cRCT supervisors and programme managers	N/A	Individual interviews

### Data collection

A round of individual interviews were conducted first, followed by the two focus groups, [26], and a subsequent round of individual interviews [27]. Interviews with programme stakeholders were conducted as a separate process [28]. Focus groups were conducted with the aim of creating additional opportunities for participants to discuss their experiences, as it was anticipated that the conversation among the participants would trigger new thoughts, thereby expanding the data [38].

Both individual and focus group interviews were conducted in a private space at a local training and research centre. Informed, voluntary consent was obtained before any data were collected. Data were collected in isiXhosa by a first language speaker of isiXhosa, and then translated and transcribed. Interviews were conducted by a data collector with extensive training in qualitative methods. The researcher (LSK) and the data collector worked closely together to ensure the quality of the data; each interview was discussed in detail immediately after completion. The data collector conducting the qualitative interviews had extensive experience conducting similar interviews.

### Data analysis

For the three individual studies that this article draws on, data were initially analysed thematically, structured by the six steps described by [39]: familiarization, generating initial codes, searching for themes, reviewing themes, defining and naming themes, and producing the report. Transcribed interviews were reviewed line by line, and a preliminary coding scheme was developed. This coding scheme was then presented to members of the team to validate and discuss the identified themes. The analysis process is further detailed in the publications [26–28].

Drawing on the principles of a qualitative evidence synthesis (QES) [40, 41], we conducted a secondary analysis of qualitative evidence, focusing specifically on empowerment. A QES allows for a review and synthesis of qualitative evidence and can enable a further understanding of the topic studied than single primary qualitative research studies can achieve. We apply these principles by synthesising evidence across the three publications described above [41].

## Results

We organized our findings deductively in the same approach adopted by Travers et al., organizing findings across the four domains of Kanter’s organizational structural components of information, resources, support, and opportunity, and mapping these domains against Spreitzer’s domains of psychological empowerment in the workplace. Travers’s matrix consists of the psychological experiences of meaning, competence, self-determination, and impact embedded under the organizational structural components in Kanter´s components [33].

### Information

Training and access to information emerged as critical for CHWs in allowing them to feel able to carry out their responsibilities. Based on the additional training received in the parent trial, CHWs reported how this training provided information needed for carrying out their work more effectively. They reported seeing their work as more meaningful, with a tangible impact in their communities. Training emerged as essential for CHW confidence, self-determination, and meaning making.

*“As we now have backpacks and scales*, *you would find if you meet with [community members]…they would say*, *‘you guys are nurses now*, *what is it that you are doing now?’ and you tell them that you are working just like the way you were taught and they would tell you that you are on a high level*, *you will be successful… Ever since we came here and came across with [NGO] we know what to do when we go out and what to do in the homes” [FGD*, *PID8]*.

Community respect and programme legitimacy was described as built on CHW skills, confidentiality, and persistence. Being able to provide a service that was lacking in the community, appear to have strengthened CHWs’ level of respect and sense of meaning.

*“They also like it when you persevere with them not that thing of insulting a person when they have done wrong. They like that we continue with them and hold their hands even if a person is falling. We always getting them up. That is why they are giving us the respect that they give us because we are the people that are doing a good job in the villages”*. [FGD 1, PID 1] [26].

The additional training appears to have added a sense of competence, self-worth, and confidence for the CHWs, and thus enhanced the sense of meaning.

*“I have gained more from [organization]*, *even when I am walking there*, *I feel like a trained nurse*, *I know I am not a nurse*, *but I do feel like a fully trained nurse because even if I am somewhere*, *I would see someone coming to me and asking to talk to me or ask about something*, *or I would see someone entering at home to get information about something about their health*, *if she brought her clinic card with*, *I would take her on the side and then tell her what to do”* [FGD, PID 8].

### Resources

A second critical domain linked to CHW empowerment was access to the practical resources needed to perform their job effectively. Shortages in equipment and transport affected CHWs’ ability to conduct their work effectively and thus directly the perceived impact, as well as their sense of competence and self-efficacy.:

*“This programme [government implemented CHW programme]*, *right. Firstly*, *I was going to start providing equipment for them where they will be able to*, *where they will work*, *be sure to work efficiently because they have everything. So that you don’t get people not doing what they’re supposed to just because she does not have the essentials to do the job.”* [Control CHW, PID C5] [27].

The lack of equipment was described as a major challenge that also directly affected CHWs’ legitimacy and credibility among their clients and broader communities. The provision of equipment through the Philani intervention appears to have enhanced CHWs’ sense of competence.

*“The supervisors used to give us hope*, *there are those of which we never had anything before*, *of the only thing we had was just a handbook*, *some sort of a notebook. We wrote everything on that*, *it looked like it’s not serious*, *but the information in those notebooks were very much important*, *so now that we have all the material it makes it easier to work and I am being recognized as a real [CHW]”.* [Intervention CHW, PID 1]

Through the intervention in the parent trial, some transportation and additional equipment was provided, which led to a sense of improved competency and self-determination.

*“There’s a huge difference because now we are the nurses in the public eye. Some people will say*, *‘come with that scale and measure*,*’ some will say ‘please come and do me the HIV test and pregnancy*, *check the child’s card if pills*, *vitamin A*, *are still sufficient for return date.’ …I mean there is a huge difference now that we have the material. And you walk with confidence knowing that you are going to work”* [Intervention CHW, PID 1].

### Support

Support emerged as critical on two levels – CHWs relied on support from the health care system, but also on community support. CHWs described how they were at times seen as volunteers in the community with no official job title, negatively affecting their credibility and sense of meaning:

*“Because they [patients] are undermining us or they take us as the doctor’s slave or servants for nurses*, *that is how they take us*, *they do not really know about our work*, *they think that we were sent by the nurses to come to them…They can undermine me*, *but at the end of the day*, *those people would call me while I am passing by and say you must please come back because there is this and that. That is the challenge that I see as hurtful*, *being undermined by your work as if you choose the work yourself*, *not knowing that God gave you this work because you are the best with this work*, *they do not even know that*, *they think that we are slaves for doctors and nurses to do their work.”* [FGD PID5].

CHWs also spoke about a lack of support from within the formal health care system. They were often being tasked with administrative activities at the clinics rather than being able to conduct their work in the communities, CHWs felt handicapped; one programmeprogramme manager identified how this practice further undermined their role.

*“I mean*, *we saw that the clinic just didn’t have enough personnel so something that we found often was that the nurses in the clinics instead of sending the CHWs out in the community would just use them to do admin stuff or basic stuff in the clinics which is to my mind absolutely a signal of a larger problem.”* [Programme manager 1] [28].

Participants described their lack of integration as harmful to their credibility,

*“To them [community members] it is like we are not employed as compared to those who are working in the clinic*, *so in that case we need to sit down with that person and explain to her about our job and try to show her the help we bring to the community”.* [FGD 2, PID 9] [26].

Programme managers described their need for an accountable, supportive system, to make it possible for CHWs to carry out their roles in demanding, often isolated environments:

*“I think what I saw in practice was that if you are appointed to do a job that you are not equipped to do in any way*, *and you have zero support and no one is there to train you*, *especially if you are working in that kind of geographical area where I mean it’s so far removed from hospitals*, *from private doctors*, *there is just nothing – and all of a sudden you’re this person who has to help people but you don’t actually know how to help them at all*, *I mean it’s incredibly discouraging.”* [Programme manager 2] [28].

This same individual also commented on how this sense of supportiveness and connectedness could be enhanced by better health system leadership, such as having a point person in the hospital setting dedicated to liaising with CHWs:

*“The support is also really poor*, *people feel that they are isolated*, *on their own*, *there is nobody who can give them advice*, *there is nobody who can tell them where the patient should go and that is what is so useful to have a link into the hospital”* [Programme manager 2] [28].

These data highlight a critical domain that emerged from our study, namely supportive supervision. It was evident that supervision was limited for all CHWs prior to the intervention implementation in the parent trial. Respondents described how the supportive supervision provided in the parent trial had positive effects on CHW motivation and self-determination.

*“I think that it’s nice to have a supervisor even though we needed to adjust to it since we were working on our own before and there were no targets or paperwork. It’s also beneficial to us because I gained a lot of knowledge and understood my work more and it becomes easier as you have a supervisor checking up on you. The organization also gives us courage to do more for our communities*, *cover more areas”.* [Intervention CHW PID 6]

*“the … training made a huge difference in my work experience because it had materials; we were trained and received the materials*, *you get trained then you also do what you were trained for*, *and clients notice that there’s a huge difference. This and that wasn’t happening before and when it’s a visit time you see that everyone is excited; that’s what gave us higher level. They see that the nurses have arrived*, *you will see other people arriving from other houses*, *the neighbours will come because they see we are working here”.* [Intervention CHW, PID 8]

### Opportunity

Finally, CHWs, managers, and stakeholders spoke to multiple unmet needs and programmatic shortfalls, providing nuanced perspectives on task-shifting, programme structure, and perceived impact.

*“Currently the community healthcare workers are unable to work because they don’t have supervisors*, *they are not permanently employed*, *they don’t have tools and they can’t go where they want to go or where they are needed. … Community health workers are uncertain of their employment and once you have job dissatisfaction you don’t get motivated or become productive because you don’t know where you fall under.* [Operational clinic manager 3] [28].

The issue of motivators, support, and opportunity also extended to considerations about the potential for impact on both individual and systems levels.

“*There is very little support for them and therefore there is very little support for the CHWs so that they are demotivated and not that effective and many of them are actually not out in the field. So my sense is that we haven’t actually worked out a system*, *a structure that works. I am worried that even if they get paid little*, *60 000 CHWs will be a big expense without a massive benefit for the health system. [Programme manager 1]*” [28].

The intervention in the parent trial appears to have facilitated opportunities for CHWs to learn and develop, and this in turn, is reported to have improved both the CHW work situation, sense of competency and meaning,

*“Babies used to die*, *but by us being here*, *we educate them that they must go to the clinic before 20 weeks to book because if you are just sitting there*, *maybe you do not know what your status is while you are sitting at home…We used to hear that children are dying*, *maybe the other one would give birth and die after*, *or they would give birth to a stillborn baby*, *but now it is better in our areas*, *the babies are being born beautiful*, *you would see that they baby is beautiful because of our teachings*, *we work in these homes*, *we are doing big things in these homes” [FGD 2*, *PID 3]*.

## Discussion

This study aimed to document barriers and facilitators to programme success through triangulating evidence from diverse stakeholders involved in CHW programme administration and delivery—and to shape next steps to improving CHW programmes. Our findings provide important considerations for CHW empowerment, and the conditions that facilitate it in the rural South African context, for policymakers and programme implementers. We discuss these findings and close with a series of emerging recommendations that could better facilitate more productive integration of CHWs into routine health services.

It is evident that the different programatic levels within CHW programming such as resources and training, and relational domains such as trust, and connectedness may facilitate CHW empowerment. Training and access to sufficient information and knowledge to perform the tasks expected is regarded as critical in facilitating the empowerment of CHWs. As illustrated in the model by Travers et al. [33], there is a direct link between access to information and empowerment, making this a pivotal step in giving CHWs control over their work which generates a sense of meaning. In our findings, the additional training facilitated this process. In the South African national CHW program, CHWs typically receive an initial 10-day training, which could be enhanced by continuous follow-up trainings, or alternatively further training provided through supervisors in the field. Similarly, access to sufficient resources such as equipment and transport is linked to their sense of competence, which is critical in facilitating empowerment. The limited access to resources suggests a lack of prioritization that led to further marginalization of CHWs. By contrast, the resources added through the intervention facilitated the sense of competence amongst the CHWs. While it may come with additional expenditure, investing in equipment such as scales, and additional access to transport, would be worthwhile even in resource-scare settings. Similarly, investments in supportive supervision are critical in facilitating programmatic improvements. In the South African national CHW program, there are specific roles for CHW supervisors, however in practice, these positions appear to often be vacant or merged with other roles, leaving little time for actual supervision. In our data, ongoing support from both the community and the formal health care system emerged as critical in facilitating CHW empowerment, as well as having access to opportunities to grow, learn and conduct work effectively. Clear challenges in these domains were identified, and the intervention in the parent trial appears to have mitigated some of these challenges through providing additional training, equipment, and supportive supervision.

Programme legitimacy was closely linked to empowerment, and CHWs described the need for access to information, support, equipment, and opportunities for career development and learning if they were to be seen as credible in the communities in which they work. CHWs who lacked access to necessary resources and support experienced reduced motivation and were unable to carry out their work effectively [42, 43]. While CHWs may begin to feel empowered through training, they require a supportive system to do so. In many cases, this supportive infrastructure is simply missing. Unless improvements are made in the programmematic domains identified, CHWs risk remaining disempowered, jeopardising the potential beneficial effects of such programmes. CHWs have a depth and breadth of knowledge of their communities that is not only unique to each community but goes beyond the usual biomedical parameters to include the social determinants of health. If they are to play a more constructive role in the health system in terms of community-oriented primary care, their expertise needs to be acknowledged and respected as important members of a collaborative team rather than at the lower end of a hierarchy [18]. Without their empowerment and mutual acknowledgement as equal partners, their expertise is lost to the health system.

Lashinger has argued that the capacity of organizations to empower staff lies in two specific sources of power in organizations [44]. These are (1) formal power (specific job characteristics); and (2) informal power (interpersonal relationships with superiors, peers, and subordinates). It is likely that investments in informal power for CHWs, which could disrupt the prevailing power relations might facilitate the process of including CHWs in decision-making relating to programme implementation. A practical strategy to ensure CHW inclusion could be to implement formal systems through which CHWs are consulted as a core part of ongoing continuous improvement efforts. This would place CHWs in a more empowered position to influence strategic decisions without any significant additional .expenditure, making the strategy attainable in resource-scarce settings.

There is extensive evidence of the wide scope of roles and tasks that CHWs deliver [45]. They often do this without sufficient training, equipment, and supportive supervision, raising concerns regarding their rights and needs [14, 46]. These roles and tasks need to be further assessed to ensure that they are acceptable for CHWs and their target audience [45]. In our study this did not happen, with the result that CHWs and stakeholders described uncertainty and the lack of clarity about roles. CHWs commonly reported being drawn into working in the clinics rather than out in the communities, to the detriment of community-based services such as home visiting. The reasons for this were largely that the clinics were short-staffed and under-resourced, challenges reported by CHWs and clinic personnel in this study and elsewhere [47, 48]. This sense of confusion negatively influenced the legitimacy of the cadre as their roles are not clearly defined and therefore may be seen as less meaningful. Further, Maes [49] discusses the importance of exploring the narratives of empowerment and includes looking at political, historical, and relational contexts. In the case of CHWs, one must take into consideration structural constraints to empowerment, such as strictly hierarchical health systems, and CHWs traditionally being economically disadvantaged women. This is also the case in this study, although two of the CHWs were male.

CHWs’ roles, situated as they are between the healthcare system and the community, are critical in facilitating trust in the health system [50]. This further underlines the importance of a functioning healthcare system that supports the CHW programme. To facilitate the empowerment and the legitimacy of the CHW cadre, however, we need a deeper understanding of the complex roles that CHWs play between these two systems [50]. Integrating CHWs within formal health care services is critical for the legitimacy of the programme and the functioning of the health system. CHWs’ position between the community and the formal health system may also pose challenges for CHWs themselves, as they are then required to traverse both worlds and have one foot in each place [51]. Our data supports the view that the rapid implementation of the national CHW programme in South Africa may have happened within a health system that was not functioning sufficiently well to fully and appropriately integrate CHWs within the system [21, 52]. Political support and sufficient investments are critical in ensuring a functional CHW programme, and insufficient prioritization can be detrimental for a national CHW programme.

Our findings reinforce the principles of the model by Travers [33], and the theory by Spreitzer [36] in that the domains of structural empowerment in the workplace are directly linked to the domains of psychological empowerment, both of which are critical for CHW empowerment, and consequently for well-functioning primary health care teams. Our findings show how CHWs are still working in the periphery of the healthcare system. Without sufficient prioritisation, high level-support from primarily national and district government, and sufficient investments in programmatic domains—such as training, equipment, and supportive supervision—it is likely that the CHW cadre will continue to be seen as informal health care workers and working on the margins. This de-prioritization directly influences their credibility. We argue that supporting and fulfilling the domains of structural empowerment for CHWs is likely to lead to psychological empowerment, which can in turn position CHWs better to navigate both the power structures within the health system, and their roles in the community. CHW empowerment could be a lever to potentially transform the current health system towards universal coverage; however, this process can only happen with sufficient high-level prioritization and investment.

### Limitations

Data were collected in isiXhosa and then translated and transcribed. The process of translation into another language, as well as the process of transcribing audio-recorded interviews into text, may have meant that some minor points were lost. By a close collaboration between the researchers and the data collector, where every interview was discussed in detail, we believe this challenge was minor. Furthermore, the data was transcribed and translated by a team with vast experience in similar interviews, and with rigorous quality control mechanisms in place. The CHWs interviewed were from a relatively small pool of CHWs taking part in the larger trial who were employed by the Department of Health. This may have caused concerns about confidentiality, and furthermore may have influenced the level of candidness in the interviews, particularly when giving critical feedback. We believe the risk was small, given the measures we used to ensure confidentiality: the participants were de-identified by using PIDs, and any names of clinics and geographical areas were removed. Furthermore, the interviewer was not known to the CHWs and was independent of the Eastern Cape Department of Health employing the CHWs. Although the sample is relatively small, critical lessons can be learnt and insights transferred to similar contexts.

### Recommendations

CHW programme legitimacy and the empowerment of CHWs are closely linked and an essential element of this is prioritising CHWs. Empowerment can be facilitated by investing in sufficient equipment, training, and supportive supervision. Investments in these areas could in resource-strained environments be: (1) adding in-house follow up trainings for CHWs, (2) adding additional low cost equipment such as scales, and (3) ensuring that the CHW supervisor has sufficient time and resources available to perform supervision in the field, and (4) regular scheduled engagements between facility-based and community-based health workers to ensure integrated service delivery to the same community. In this way, CHW programmes can bridge the gap between facility-based services and community-based initiatives in terms of community-oriented primary care, towards universal health coverage.

CHWs and supervisors have invaluable knowledge of the community and context in which they work, and their knowledge should be used in the design and implementation of programmes [26, 53]. When their perspectives are sought, they are often post-intervention, involving questions about training and implementation obstacles. Their viewpoints should be solicited prospectively in programme design. Future CHW programme efforts could benefit from adopting a co-production approach in the programme design engaging different stakeholders to work together to develop or improve services [53]. Co-production of implementation processes could also facilitate the process of structural empowerment and thus CHWs’ sense of empowerment, one key component in the greater push for universal health coverage.

It is evident that the relation between the formal healthcare system and CHWs is complex. It is critical that CHWs are fully included as equal partners in the healthcare system and have clear roles and mutually respectful relationships with healthcare professionals. A first step in achieving this would be to clarify the CHW role within the health system and thus also ensure an awareness of both CHW roles, and the benefits of CHWs. Ensuring that CHWs are fully included in the primary health care teams at a local level is critical, including CHWs in clinic meetings and strategy development activities could be ways to do this. Clarifying roles and fulfilling the domains of structural empowerment would facilitate this process and equip CHWs to better navigate the healthcare systems.

## Conclusion

While this study was conducted in South Africa, findings are applicable to other countries experiencing high inequality and are particularly relevant for other LMICs. Facilitating empowerment of CHWs is critical for the legitimacy of the cadre. Fulfilling the domains of structural empowerment through ensuring sufficient access to information, resources, support and opportunities, a process of psychological empowerment can happen. By thus bolstering the confidence and competence of CHWs, they could fulfil a critical role between health services and the communities that they serve, in achieving universal health coverage.

### Electronic supplementary material

Below is the link to the electronic supplementary material.

Supplementary Material 1

## Data Availability

The datasets used and/or analysed during the current study are available from the corresponding author on reasonable request.
