# Supplementary material for: From the periphery to inclusion within the health system: promoting community health worker empowerment as a way forward
Source: BMC Prim Care. 2024 Jul 26;25:272. doi: 10.1186/s12875-024-02523-0 (PMC11282798; doi:10.1186/s12875-024-02523-0)
Supplement: Supplementary file 1 — Supplementary Material 1 [file 12875_2024_2523_MOESM1_ESM.docx]

**Supplementary file: Interview guides**

1. **Interview guide CHWs**

- **Tell me about yourself.**
- Full name/current village/current age, where originally from, other places lived
- Highest education completed? Including post-graduate training/any informal community training, what kinds of prior jobs or experiences
- For how long have you been a CHW?

Working as a CHW

- **Why did you decide to become a CHW?**
  - What kind of things about the job appealed to you? What made you nervous?
- **What is a CHW? How would you describe it in a few sentences?**
- **What do you like the most about being a CHW and why?**
- **What do you like the least about being a CHW and why?**
- **What is the most important part of being a CHW?**
- **What are the main challenges you meet in your job as a CHW?**
- **What is it like to work in your own community, perhaps with people you know?**
- **Do you ever feel burdened or stressed over your job?**
- **What do you think about the CHW programme in general? What are the most important parts of the programme?**
- **Do you think home visiting is a good way for a programme to work?** In what ways? Do you think other women in your area, or other parts of South Africa, may benefit from it?
- **If you could change/improve something about the CHW programme what would you do differently?**
- **Do you ever think about your clients’ problems after a visit/after your workday is done?**

What kinds of problems or situations stick with you the most?

- **Do you think this job has had a noticeable impact on your own life? In what ways?**

Good (financial, emotional, social, confidence) or bad (distress, pressure, uncertainty)

The Philani home visiting model

I am now going to ask you a few questions regarding how your job has changed since you took part in the Philani training and started working with the Philani intervention model. By the Philani model we mean using folders and phones, weighing all children, using everything you learnt at the training, and having new supervisors who visit you in the field every 2 weeks.

- **Please tell me about how you experienced the Philani training?**
  - What new skills did you learn?
  - What was enjoyable?
  - What was challenging?
  - Which of the skills you learnt in the training are you using in the field when you work?
- **Please tell me about the difference between your role as a CHW previously and your role now (after joining the Philani training)?**
- **Please tell me how your job has changed since you took part in the Philani training and started working with the Philani intervention model.**
  - Is it more enjoyable of more challenging? Explain
  - Has the way you interact with your clients changed? Explain
  - Has your work load changed? Explain
- **Thinking back to what you explained being challenging with your job, has this changed at all since you started working with the Philani system? Explain**
- **Do you think anything needs to change to make the programme work better?**
- **What big challenges are there in this particular area that make program success difficult?**
  - Do you feel like the community where you are working is being helped by this intervention? What do you notice?
  - Have you experienced any negative community reactions to the programme, before or after the Philani system was introduced?

Supervision:

Remember, some of what we discuss may be sensitive, but it is all confidential and won’t be shared with anyone on the team, especially your supervisors.

- **Tell me about the supervisors – how do they work with you?**
  - How much contact do you have with them, what do they do when on a visit with you?
- **Do you think you have enough support from your supervisors? What would you change?**
- **What was supervision like before you joined the Philani training?**
- **If and how has supervision changed since you did the Philani training? Explain the differences**
  - **Have you had any experience problem solving for a client with your supervisor?**
- **Have there been any problems, conflicts, or concerns arising between you and any of the supervisors or management staff? Tell me about it.**
- **How has using a mobile phone to log your home visits been?**
  - Benefits
  - challenges
    - - **How would you describe your relationships with other CHWs**
      - Do you support each other as CHWs? How?
      - Do you ever discuss your challenges in an unstructured way? (On phone/after work)

Relationships with mothers:

Now I am going to ask a little about your relationships with your clients.

- **What are some strategies you have used to build connections with your clients?**
  - Do you think these are effective strategies? How so?
- **What makes you connect with one client better than another, in your opinion?**
  - What challenges do you face when you are getting to know a client?
- **Do you notice your clients all following the advice or information that you give them? What kinds of things do you see?**
- For those who don’t – why do you think they aren’t following your advice?

# **Interview guide: Focus groups**

- **Tell me about yourselves**
- Full name/current village/current age, and how long you have been a CHW for

Working as a CHW

- **What is the most important part of being a CHW?**
- **What are the main challenges you meet in your job as a CHW?**
- **What do you think about the CHW programme in general? What are the most important parts of the programme?**
- **Do you think home visiting is a good way for a programme to work?** In what ways? Do you think other women in your area, or other parts of South Africa, may benefit from it?
- **If you could change/improve something about the CHW programme what would you do differently?**

The Philani Intervention model

I am now going to ask you a few questions regarding how your job has changed since you took part in the Philani training and started working with the Philani Intervention model. By the Philani model we mean using folders and phones, weighing all children, using everything you learnt at the training, and having supervisors who visit you in the field every 2 weeks.

- **Please discuss how you experienced the Philani training?**
  - What new skills did you learn?
  - What was enjoyable?
  - What was challenging?
  - What are you using in the field when you work?
- **Please discuss the differences between your role as a CHW previously and your role now when you are working with the Philani Intervention model?**
- **Please discuss how your job has changed since you took part in the Philani training and started working with the Philani Intervention model?**
  - Is it more enjoyable of more challenging? Explain
  - Has the way you interact with your clients changed? Explain
  - Has your work load changed? Explain
- **Thinking back to what you explained being challenging and what was being awarding with your job, has this changed at all since you started working with the Philani model? Explain**
- **Do you think anything needs to change to make the programme work better?**
- **What big challenges are there in this particular area that make program success difficult?**
  - Do you feel like the community where you are working is being helped by this intervention? What do you notice?
  - Have you experienced any negative community reactions to the programme, before or after the Philani system was introduced?

Supervision:

- **Tell me about the supervisors – how do they work with you?**
  - How much contact do you have with them, what do they do when on a visit with you?
- **Do you think you have enough support from your supervisors? What would you change?**
- **What was supervision like before you joined the Philani training?**
- **If and how has supervision changed since you did the Philani training? Explain the differences**
  - **Have you had any experience problem solving for a client with your supervisor?**
- **Have there been any problems, conflicts, or concerns arising between you and any of the supervisors or management staff? Tell me about it.**
- **How has using a mobile phone to log your home visits been?**
  - - - **How would you describe your relationships with other CHWs**
      - Do you support each other as CHWs? How?
      - Do you ever discuss your challenges in an unstructured way? (On phone/after work)

Relationships with mothers:

Now I am going to ask a little about your relationships with your clients.

- **What are some strategies you have used to build connections with your clients?**
  - Do you think these are effective strategies? How so?
- **What makes you connect with one client better than another, in your opinion?**
  - What challenges do you face when you are getting to know a client?
- **Do you notice your clients all following the advice or information that you give them? What kinds of things do you see?**
- For those who don’t – why do you think they aren’t following your advice?

# **Interview guide: Supervisors**

- **Tell me about yourself.**
- Full name/current village/current age, where originally from, other places lived
- Highest education completed? Including post-graduate training/any informal community training, what kinds of prior jobs or experiences

Working as a supervisor

- **Why did you decide to become a MM/CHW supervisor? Tell me about your career path**
- **For how long have you been a MM/CHW supervisor and for how long were you an MM before that?**
- **What are your responsibilities as a supervisor?**
- **What do you like the most about being a CHW supervisor and why?**
- **What do you like the least about being a CHW supervisor and why?**
- **What is it like to work in your own community, perhaps with people you know?**
- **Do you ever feel burdened or stressed over your job?**
- **What is the most important part of being a CHW supervisor?**
- **What are the main challenges you meet in your job as a CHW supervisor?**
- **What do you think about the CHW programme in general? What are the most important parts of the programme?**
- **Do you think home visiting is a good way for a programme to work?** In what ways? Do you think other women in your area, or other parts of South Africa, may benefit from it?
- **If you could change/improve something about the CHW programme what would you do differently?**
- **Do you ever think about your clients’ problems after a visit/after your workday is done?**

What kinds of problems or situations stick with you the most?

- **Do you think this job has had a noticeable impact on your own life? In what ways?**

Good (financial, emotional, social, confidence) or bad (distress, pressure, uncertainty)

Supervising CHWs

I am now going to ask you a few questions regarding your experience of supervising already trained and working CHWs as opposed to newly recruited ones.

- **What has been the most challenging thing with supervising CHWs in this project?**
- **What have you enjoyed the most?**
- **Please tell me about the difference between your role as a supervisor previously and your role within this project.**
- **Please tell me about what it has been like to supervise the CHWs in this project from the beginning of the project?**
  - Challenges
  - Progress
- **Please tell me about the supervision strategies you are using at the moment and how they are working**
- **How has it been using mobile phones a supervision strategy?**
  - Benefits
  - Challenges
- **Please tell me about your relationships with the CHWs**
- **Do you think anything needs to change to make the programme work better?**
- **What big challenges are there in this particular area that make program success difficult?**
  - Do you feel like the community where you are working is being helped by this intervention? What do you notice?
  - Have you experienced any negative community reactions to the programme, before or after the Philani system was introduced?

# **Interview guide: Clinic personnel**

- Please describe your role
  - What is your experience of CHW programmes?
  - Context of work?
  - How did you get into role?
  - Motivation behind it?
  - When did you start your current role?
- Please describe the CHW program that you are part of, or that is happening at your clinic currently?
  - Program design
  - Funding
  - Where/how
  - How does supervision work?
- What are some challenges and successes with your current CHW program
  - Typical challenges
  - Challenges for CHWs in this area
  - COVID, long term? challenges
  - Strikes
  - Protests
  - Do CHWs mostly work in the field or in the clinic?
- If you were to run the CHW program, what would you do differently?
- The CHWs from your clinic were part of the Philani training in 2017. Did anything change in the way they work after the training?
- **For intervention clinic personnel**: CHWs at your clinic have been supervised by Philani since 2017
  - How did the training affect the CHWs work?
  - How did the supervision affect CHWs work?
  - Positives and negatives with the program?

# **Interview guide: Program managers**

- Please describe your role
  - What is your experience of CHW programmes?
  - Context of work?
  - How did you get into role?
  - Motivation behind it?
- Please describe the CHW program that you are managing currently?
  - Program design
  - Funding
  - Where/how
  - How do you manage the program? E.g in the field/local office/head office?
- What are some challenges and successes with your current CHW program
  - Typical challenges
  - Challenges for CHWs in this area
  - COVID, long term? challenges
  - Strikes
  - protests
- Please elaborate on the current status of CHW programmes and what are your thoughts for the future? South African/African/Global
- What, in your opinion is keys to successful CHW programmes/what are the needs for successful CHW programmes and how do we get there?
- There appear to be many issues with CHW programmes, particularly when they are scaled up – how do we fix this?
- CHW programmes in other countries (e.g India) appear to be more successful that our one in SA – why do you think that it?
- CHW used to have a vertical approach (focusing on one issue only), now it is horizontal – do you think this is good/bad why?
- Im interested in government run vs NGO run programmes - have you got experience in one or both?
  - Please discuss your experience of NGO vs government run CHW programmes?
- If we apply a more general, almost philosophical lens – what are your thoughts on CHW programmes – are they the right way to go? Is home visiting working?
- Please share your thoughts on evidence-to-practice and what makes this especially challenging. E.g. – what do we know and how should we use that knowledge?
- Broader application/growth of these programmes – how do we grow these programmes in a sustainable way, keeping/increasing efficacy? From knowledge to practice – data? But then?
- Lastly, I would like to know more about your experiences of supervision in CHW programmes. Please describe your experiences.
  - how important is supervision?
  - keys to successful supervision?
  - What are the issues with current supervision?
  - What is required for successful supervision? Examples of models
